# Antenatal tobacco use and iron deficiency anemia: integrating tobacco control into antenatal care in urban India

**DOI:** 10.1186/s12978-018-0516-5

**Published:** 2018-05-02

**Authors:** Ritesh Mistry, Andrew D. Jones, Mangesh S. Pednekar, Gauri Dhumal, Anjuli Dasika, Ujwala Kulkarni, Mangala Gomare, Prakash C. Gupta

**Affiliations:** 1 Department of Health Behavior and Health Education, University of Michigan School of Public Health, 1415 Washington Heights, SPH I, Room 3806, Ann Arbor, MI 48109-2029 USA; 2 Department of Nutritional Sciences, University of Michigan, Ann Arbor, USA; 3 Healis Sekhsaria Institute for Public Health, Navi Mumbai, India; 4 Municipal Corporation of Greater Mumbai, Mumbai, India

## Abstract

**Background:**

In India, tobacco use during pregnancy is not routinely addressed during antenatal care. We measured the association between tobacco use and anemia in low-income pregnant women, and identified ways to integrate tobacco cessation into existing antenatal care at primary health centers.

**Methods:**

We conducted an observational study using structured interviews with antenatal care clinic patients (*n* = 100) about tobacco use, anemia, and risk factors such as consumption of iron rich foods and food insecurity. We performed blood tests for serum cotinine, hemoglobin and ferritin. We conducted in-depth interviews with physicians (*n* = 5) and auxiliary nurse midwives (*n* = 5), and focus groups with community health workers (*n* = 65) to better understand tobacco and anemia control services offered during antenatal care.

**Results:**

We found that 16% of patients used tobacco, 72% were anemic, 41% had iron deficiency anemia (IDA) and 29% were food insecure. Regression analysis showed that tobacco use (OR = 14.3; 95%CI = 2.6, 77.9) and consumption of green leafy vegetables (OR = 0.6; 95%CI = 0.4, 0.9) were independently associated with IDA, and tobacco use was not associated with consumption of iron-rich foods or household food insecurity. Clinics had a system for screening, treatment and follow-up care for anemic and iron-deficient antenatal patients, but not for tobacco use. Clinicians and community health workers were interested in integrating tobacco screening and cessation services with current maternal care services such as anemia control. Tobacco users wanted help to quit.

**Conclusion:**

It would be worthwhile to assess the feasibility of integrating antenatal tobacco screening and cessation services with antenatal care services for anemia control, such as screening and guidance during clinic visits and cessation support during home visits.

## Plain english summary

Tobacco use and anemia are harmful to pregnant women and their fetuses. In India, tobacco use during pregnancy is not addressed when woman receive antenatal care, while anemia and other nutritional problems, which are more common, make up key components of care. We studied the relationship between tobacco use and anemia to find opportunities to integrate tobacco cessation into antenatal care at governmental health centers in Mumbai, India. We interviewed patients and did blood tests for tobacco use, anemia and iron-deficiency. We also interviewed providers and had focus group discussions with community health workers who worked at antenatal care clinics.

We found that 16% of patients used tobacco, 72% were anemic, 41% had iron deficiency anemia (IDA) and 29% were food insecure. Tobacco use dramatically increased the risk of IDA and consumption of green leafy vegetables lowered the risk. Tobacco users compared to non-users were just as likely to consume iron-rich foods and to be food secure. Clinics had a system for screening, treatment and follow-up care for anemic and iron-deficient patients, but no services were present for tobacco use. Clinicians and community health workers were interested in integrating tobacco screening and cessation services with current maternal care services such as anemia control. Tobacco users wanted help to quit. It would be worthwhile to assess the feasibility of integrating antenatal tobacco screening and cessation services with antenatal care services for anemia control, such as screening and guidance during clinic visits and cessation support during home visits.

## Background

The rate of tobacco use among adult women and pregnant women in India is about 10% with widely varying rates by region [1, 2]. Despite the harms associated with tobacco use, tobacco cessation services are not routine parts of reproductive health care, including antenatal care. In contrast, the prevalence of anemia in India is 65% [3], and anemia prevention and control are key components of antenatal care services [4]. The National Health Missions’ (NHM) guidelines require every pregnant woman be tested for anemia and iron deficiency, and anemic women be given a free 90-day supply of iron folic acid (IFA) tablets. The NHM uses antenatal clinics and a cadre of community health workers (CHWs) called ASHA workers (Accredited Social Health Activist) to provide pregnant women relevant healthcare, education and support. In contrast, even though many women in India use tobacco even during pregnancy, there is no national scheme to address antenatal tobacco use, despite international recommendations [5].

Antenatal tobacco use and anemia appear to be correlated [6, 7], and are important risk factors for poor pregnancy outcomes [8–11]. Biochemically, tobacco use may affect iron metabolism [12], iron stores [13], inflammation [14], and hemoglobin levels [6]. Behaviorally, tobacco use may act as an appetite suppressant [15–17], and has been linked with lower food intake and household food insecurity [18–20], while tobacco abstinence may increase appetite [16, 21]. It is unclear how the relationships between tobacco use, anemia and iron status may be relevant to the delivery, uptake and efficacy of tobacco cessation services during pregnancy. We examined the links between tobacco use, anemia and iron deficiency anemia in the context of antenatal care in Mumbai, India to explore ways to integrate tobacco control services into routine antenatal care services.

In observational studies of clinical settings in India, tobacco use screening and motivational guidance to quit have been reported [22–24]. In a study of pregnant women, patients were screened [25] and users were monitored and counseled about the harms of tobacco use on the mother and fetus, but there was no effect on cessation rates. Also in a clinical setting, an observational study of tuberculosis patients used a brief application of 5-A’s model (Ask, Advise, Assess, Assist and Arrange) to help tobacco users to quit and showed promise [26].

From a community-based perspective, CHWs at antenatal clinics can help address antenatal tobacco use during home visits and community events. Their health promotion activities involve frequent contact with pregnant women at home, including coordination with antenatal care visits. When employed to address antenatal tobacco use, CHW strategies appear feasible in observational studies from India [25, 27] and elsewhere in the region [28]. One cluster randomized trail in low-income communities in India [29], used a brief (2 session) CHW delivered intervention and showed a small positive effect, a 2% improvement in cessation rates.

The current study, conducted at urban governmental Primary Health Centers (PHCs) aimed to: 1) measure the association between tobacco use and anemia; 2) assess the role of iron-rich food consumption and household food insecurity; 3) identify ways to integrate tobacco cessation services with existing antenatal care services. We hypothesized that tobacco use would be strongly associated with anemia and iron deficiency anemia and consumption of iron-rich foods and household food insecurity would be associated with tobacco use, anemia and IDA. We identified opportunities to address tobacco use as part of antenatal anemia care.

## Methods

### Design

This observational study was conducted in Mumbai, India at antenatal clinics in 5 PHCs. We recruited 100 pregnant women (20 per clinic) from waiting areas by approaching all patients. A research staff person recruited patients, screened for eligibility, and enrolled those who gave informed consent. Inclusion criteria were: receiving antenatal care at the PHCs; first or second trimester; no major pregnancy complications; and age 18–45. We also recruited 75 antenatal care providers from the five clinics (five doctors, five auxiliary nurse midwives (ANMs), and 65 CHWs). Current antenatal providers at the PHCs were eligible. Data were collected from April 2015 to April 2016.

### Measures

#### Pregnancy questionnaire

We conducted 60-min face-to-face structured interviews in *Marathi* with pregnant women in private settings after antenatal care appointments.

#### Tobacco use

Tobacco use items were adapted from the Global Adult Tobacco Survey [30]. Current tobacco use was defined as past 30-day use of smoked / chewed / applied tobacco. Amount of tobacco use was measured as number of days and number of times per day in the last 30 days tobacco was smoked / chewed / applied. We measured reasons for using tobacco (helps with morning motions, satisfies craving, helps to relax, etc.); desire to quit and to receive cessation counseling; and antenatal cessation services received (screening, cessation guidance and referral).

#### Consumption of iron-rich foods

We administered a 31-item food frequency questionnaire to assess past 90-day consumption of iron-rich foods, vitamin A and folate as well as foods high in facilitators (e.g., ascorbic acid) and inhibitors (e.g., tannins) of iron absorption. The food list was adapted from a previously validated tool used in a similar context to our study population [31]. We measured weekly beef, lamb, chicken (meat/poultry) consumption by taking the median frequency value (0.35) for these items and grouping the sample into *no weekly consumption* (0), *less than median weekly consumption* (< 0.35) *or more than median weekly consumption* (≥ 0.35). A similar approach was used to categorize weekly consumption of fenugreek greens, spinach, black-eyed pea greens, and mustard greens (dark green leafy vegetables). The median value was 0.5 times a week. Because only one respondent reported no weekly consumption of green leafy vegetables, we combined that group with the “less than median weekly consumption” group.

#### Iron supplementation

Women were asked if they currently take an iron-folic acid tablet (Yes/No).

#### Household food insecurity

Household food insecurity (HFI) was measured using the Household Food Insecurity and Access Scale (HFIAS) [32], which includes nine Yes or No questions focused on three dimensions of household food access—anxiety about food access, food quality and food quantity—and a series of sub-questions to define the frequency of experiencing conditions linked to these three dimensions. We categorized HFI into four levels (i.e., food secure, mildly food insecure, moderately food insecure, and severely food insecure) based on standard protocols [32]. For the purpose of data analysis, we combined the moderately and severely food insecure categories because some cell sizes were small.

#### Socio-demographics covariates

We measured age, education (none, primary school, middle school, secondary school, college graduate, post-graduate), employment in the past 12 months, wealth index of household assets [33], religion (Hindu, Muslim or other) and parity (zero, one, two or more).

### Blood tests

All blood tests were done as part of routine antenatal care clinic visits. Blood samples were stored and tested by labs used by the PHCs. In total, 9 mL of blood was drawn from each participant. Portable Hemocue photometers (Hemocue, Inc., Brea, CA) were used to assess hemoglobin (Hb). Serum cotinine and serum ferritin (SF) were assessed using Chemiluminescence Immunoassay and Chemiluminescent Microparticle, respectively. Cutoffs were as follows: tobacco use (cotinine ≥15 ng/mL), anemia (Hb < 110 g/L), iron deficiency (SF < 15 μg/L) and IDA (SF < 15 μg/L and Hb < 110 g/L).

### Key informant interviews and focus groups

We conducted Key Informant Interviews (KIIs) with physicians and auxiliary nurse midwives (ANMs) and Focus Group Discussion (FGDs) with CHWs to determine the content of services provided during antenatal clinic and home visits. We asked to what extent anemia and tobacco use were evident among antenatal clinic patients; the content of anemia, IDA and nutrition-related services; how tobacco use is identified; what tobacco cessation services were provided; and how tobacco cessation could fit into the existing structure of care? We asked about the role of clinicians and CHWs in antenatal care including about anemia and tobacco use. We audiotaped all interviews and FGDs with prior permission of respondents.

### Data analysis

Quantitative data analysis was conducted using Stata 12.1 [34]. First, we examined the distribution of study variables to characterize the sample with respect to socio-demographic factors and other measures. Secondly, because we examined both self-reported tobacco use and serum cotinine we measured the sensitivity and specificity of the self-reported use against the serum cotinine cut-off described above. Sensitivity was defined as the proportion of self-reporting tobacco users who had cotinine levels at or above the cut-off. Specificity was defined as the proportion of women not reporting tobacco use who had cotinine levels below the cut-off. Third, in order understand the role of iron-rich food consumption and food insecurity, we measured the bivariate association (chi-squared test or Fisher’s exact tests when cell sizes were < 5) between tobacco use based on serum cotinine levels (*n* = 16, cotinine ≥15 ng/mL), anemia, IDA, IFA supplementation, consumption of iron-rich foods and HFI. Fourth, we used logistic regression to measure the association between IDA and tobacco use based on serum cotinine (n = 16, cotinine ≥15 ng/mL), controlling for IFA supplementation, consumption iron-rich foods, HFI and socio-demographic factors. We used the Stata’s *cluster* subcommand to account for nesting of pregnant women within clinic sites. Finally, we measured the Variance Inflation Factor (VIF) for each covariate to identify multicollinearity in our regression model [35].

We used a standard approach to conduct qualitative data analysis [36, 37]. In addition to the written notes taken during qualitative interviews and FGDs, the audio recordings were transcribed verbatim. The notes and transcripts were reviewed and discussed by members of the research team. We identified themes based on our discussions about the data. In our effort to accurately make sense of the qualitative data, we corroborated our interpretations throughout the data analysis process by checking with all members of the research team, including the field investigators.

## Results

All antenatal care patients were married, mostly young, educated up to secondary school, and lived in low-income households (Table 1). The sample was 66% Hindu and 22% Muslim. There were high levels of anemia (72%), iron-deficiency (44%), and IDA (41%). No one reported past 30-day smoking tobacco use, but 13% reported past 30-day smokeless tobacco use. In contrast, 16% had cotinine levels indicating tobacco use. The sensitivity and specificity of self-reported tobacco use was 92.3% and 95.4%, respectively. *Mishri*, a form of dried and powdered tobacco rubbed on gums, was the most common form of tobacco used (54%). Every tobacco user reported using 20 or more days out of 30 days, and 69% used two to five times a day. The top reported reasons for using tobacco were: helps with morning motions (46%), satisfies a craving (46%), enjoy using (38%), and helps when upset or relaxes (23%). One (8%) woman said she used when feeling hungry. Ninety-two percent wanted to quit, and 77% wanted help to quit. Tobacco use was not well addressed during antenatal care visits; only two tobacco-using women were asked about tobacco use and advised to quit.

**Table 1:** Characteristics of pregnant participants (*n* = 100)

	number of participants
Age in years (mean = 25.5; SD = 0.5)	
18–19	7
20–29	72
30–39	18
40–41	1
Missing	2
Parity	
0	43
1	32
2 or more	25
Currently Married	100
Education	
None	6
Primary or middle school (grade 1–7)	22
Secondary school (grade 8–12)	62
College or more	10
Monthly household income	
Less than 10,000 INR	39
10,001–15,000 INR	43
More than 15,000 INR	15
Don’t Know	3
Employed in the last 12 months	
Yes	28
No	71
Missing	1
Religion	
Hindu	66
Muslim	22
Other	12
Current smoking tobacco use (self-report)	0
Current smokeless tobacco use (self-report)	13
Current tobacco use (serum cotinine^a^)	16
Anemia^b^	72
Iron deficiency^c^	44
Iron deficiency anemia^d^	41

^a^ cotinine≥15 ng/dL

^b^ hemoglobin< 110 g/L

^c^ ferritin< 5 mg/L

^d^ hemoglobin< 110 g/L & ferritin< 15 mg/L

Tobacco user had higher rates of anemia (*p* = .13 and IDA (*p* < .05) than non-using pregnant women (per cotinine blood tests) (Table 2**)**. There was no statistically significant difference in past 30-day intake of IFA supplementation among those who used (33%) and those who did not (24%) (*p* > .05). Green leafy vegetable consumption was less common in tobacco users (31%) than non-users (52%), but the difference was not statistically significant (*p* = .12). Nearly one-third of households were food insecure (29%), but HFI was not associated with tobacco use status or IDA. None of the tobacco users reported ‘mild’ food insecurity, while 12% of non-users did. Table 3 shows that IDA was strongly associated with tobacco use (OR = 14.3, 95% CI [2.6, 77.9]), and negatively associated with weekly meat (OR = 0.1, 95% CI [0.03, 0.6]) and green leafy vegetable consumption (OR = 0.6, 95% CI [0.4, 0.9]), and not associated with HFI. There was no indication of multicollinearity in our regression analysis (VIF mean = 1.47, range [1.17, 2.28]).

**Table 2:** Distribution of antenatal anemia, iron status and other nutritional factors by tobacco use and iron deficiency anemia (*n* = 100)

	Percent				
	Tobacco use^d^		Iron deficiency anemia^c^		Overall Sample (*n* = 100)
	User	Non-user	Yes	No	
Anemia^a^	^e^88	69	.	.	72
Iron deficiency^b^	**69	40	.	.	44
Iron deficiency anemia^c^	**69	36	.	.	41
Taking iron / folic acid supplements	33	24	23	27	25
Meat / Poultry Consumption					
None (0)	13	19	*27	12	18
Less than median	50	42	46	52	50
More than median	38	39	27	36	32
Green Leafy Vegetable Consumption					
Less than median	^f^69	48	46	54	65
More than median	31	52	54	46	34
Household Food Insecurity					
Food secure	75	70	71	71	71
Mildly food insecure	0	12	10	10	10
Moderate or severely food insecure	25	18	20	19	19
Overall Sample	*16*	*84*	*41*	*59*	*100*

^a^ hemoglobin< 110 g/L

^b^ ferritin< 15 mg/L

^c^ hemoglobin< 110 g/L & ferritin< 15 mg/L

^d^ cotinine≥15 ng/dL

^e^ *p* = 0.13

^f^ *p* = 0.12

* *p* < 0.10, ** *p* < 0.05

Note: Since only one person reported no weekly consumption of green leafy vegetable, she was grouped with the less than median category

**Table 3:** Logistic regression of maternal iron deficiency anemia^a^ (*n* = 98)

	OR	95% CI
Tobacco use^b^	**14.3	(2.6, 77.9)
Taking iron-folic acid supplements	0.6	(0.1, 4.0)
Meat Consumption		
None (0)	Referent	–
Less than median	**0.3	(0.1, 0.7)
More than median	**0.1	(0.03, 0.6)
Green Leafy Vegetable Consumption^c^		
None or less than median	Referent	–
More than Median	*0.6	(0.4, 0.9)
Household Food Insecurity		
Food secure	Referent	–
Mildly food insecure	2.3	(0.4, 11.5)
Moderate or severely food insecure	1.6	(0.7, 3.8)

^a^ hemoglobin< 110 g/L & ferritin< 15 mg/L

^b^ cotinine≥15 ng/dL

^c^ There was only one individual in the “None” category, which was therefore combined with “Less than median” category

* p < 0.05

** *p* < 0.001

Note: Adjusted for age, education, employment, wealth index, religion and parity

### Key informant interviews with physicians and nurses

There was no systematic approach to address tobacco use during antenatal care at the PHCs. Services for anemia screening, treatment and supportive services were provided during clinic visits by nurses and doctors and home visits by CHWs. Clinics conducted anemia screenings at every visit and provided appropriate treatment, which included iron supplementation, guidance about nutrition, and other services as needed. Anemic patients were referred to a CHW program. Antenatal anemia was of high priority. Antenatal tobacco use was seen as a concern but not as common in the patient population. There were no systematic services in place for tobacco use screening and cessation. Providers advised known tobacco users to quit, but were not trained to provide cessation guidance and had nowhere to refer patients for cessation services, which were said to be offered at addiction centers. Providers wanted to learn about integrating tobacco use screening and cessation guidance into their routine practices. There was some concern about competing health priorities.

### CHW focus groups

CHWs were provided with a list of pregnant mothers from the PHCs, including anemia status. Their main role was to provide supportive services for anemia control during home visits that include dietary guidance, provision of and guidance about taking IFA supplementation, and reminders for follow-up clinic visits. Anemia during pregnancy was seen as a major problem. CHWs were trained to identify signs of anemia, provide advice about taking IFA tablets and offer dietary advice to pregnant women about eating iron-rich foods. They also helped women address any logistical barriers to accessing antenatal care and delivery care.

There was variability in the perception that tobacco use was a problem. At an FGD in one clinic, CHWs reported that 40% of pregnant women used tobacco, while tobacco use was not perceived as a problem at an FGD in a different clinic. CHWs do not provide any tobacco control services. They had some knowledge about the harms of smoking tobacco use, but not specifically about harms during pregnancy. Smokeless tobacco use was not universally seen as harmful. Some CHWs noted that they used fear tactics to encourage users to quit. In addition, CHWs did not receive training to deliver cessation guidance and support, but wanted to be trained, particularly if they were paid to deliver services.

## Discussion

We hypothesized that tobacco-using pregnant women would have lower consumption of iron-rich foods (i.e. meats and green leafy vegetables) and higher household food insecurity, and therefore would be at increased risk of anemia and IDA. This hypothesis was not fully supported. The regression analysis suggested that there were independent associations between IDA and tobacco use as well as consumption of iron-rich foods (meats and green leafy vegetables), but not household food insecurity. The rate of anemia was higher in tobacco users than non-users but the difference was not statistically significant, though almost every tobacco-using pregnant women was anemic. In addition, tobacco-users compared to non-users tended to eat less than the median frequency of green leave vegetable consumption. There was no notable difference in meat consumption between users and non-users.

We also hypothesized that tobacco use would be more common among individuals from food insecure households, assuming that tobacco was used to increase satiety/lower appetite, and would therefore help cope with psychological effects of food insecurity. We found no evidence that HFI was associated with tobacco use or IDA. None of the tobacco users reported ‘mild food insecurity’, that is, anxiety about food access, while 12% of non-users did. In addition, only one (8%) participant reported that tobacco was used to address hunger, while 23% said they used when upset or for relaxation.

We found that almost nine out of ten tobacco users were anemic and that tobacco use was strongly, positively and independently associated with IDA. This has important implications for antenatal care visits. In low- and middle-income countries, antenatal care, particularly anemia control services, are missed opportunities for addressing antenatal tobacco use. Tobacco control services could parallel and be integrated with anemia control services, because nearly all tobacco users appear to be anemic and would therefore receive services for anemia. Providers in our study did not screen for tobacco use, but sometimes provide quit guidance if they notice use (e.g. stained teeth or gums). They said that tobacco use screening and cessation services at clinics and home visits would be useful and feasible, especially if they integrated with current practices. Given the link between tobacco use and iron deficiency anemia found in our current study and elsewhere [6, 12–14], when discussing harms of tobacco with patents antenatal care providers can inform anemic tobacco users that their use may worsen anemia.

The integration of antenatal tobacco cessation services could face many challenges. First, CHWs and clinicians are not currently trained to look for and address tobacco use in their patients. There is a need to increase awareness about the prevalence and risks associated with antenatal smokeless tobacco use (including the increased risk of anemia and IDA) as well as to increase the clinics capacity to provide evidence-based cessation guidance and support. Unfortunately, the literature about effective antenatal *smokeless* tobacco cessation strategies is sparse, particularly in India. Second, the antenatal care system appears burdened. Competing demands was an important concern raised by providers. Antenatal tobacco cessation services should not be resource intensive, and should be well integrated with current practices. Third, the rate of current IFA supplementation in anemic women is already low at 25%, while the rate of anemia and IDA is high. This indicates that the current approaches to address anemia and IDA need improvement, and may portend a relatively small impact on antenatal tobacco use, if parallel tobacco use screening and cessation services are added. Finally, in an environment of scarce resources, additional funds will be required, particularly for capacity building activities and labor costs.

### Strengths and limitations

The key strength of this study is the use of multiple data sources (questionnaires administered to patients, biomarkers from blood tests, and qualitative information from providers) to make a careful assessment of tobacco use, anemia and iron deficiency in pregnant. Although we collected data from multiple sources, our sample size of pregnant women was not large, but it was sufficient to detect moderate effects. Smaller effects, such as those found for the association between food insecurity and tobacco use (odds ratios between 1.6 and 2.3), we not detected. The data were cross-sectional, and the observed relationships between indicate direction rather than magnitude of association or causation. The self-reported measures of tobacco use, dietary practices, IFA supplementation and HFI are prone to social desirability and recall bias. However, measures of this type are routinely used in public health surveillance systems. We used biomarkers to supplement the self-report measures. For example, our self-reported measure for tobacco use had very good but not perfect sensitivity and specificity when compared to our serum cotinine cut-off. Our cut-off was higher (≥15 ng/ml) than recommended to identify smokeless tobacco use (> 5 ng/ml) [38, 39], which was the only form of tobacco used by the participants. Our budget limited our ability to use a more sensitive blood test for cotinine. It is likely that the true rate of tobacco use in our sample was higher than 16%.

## Conclusions

We found that antenatal tobacco use was strongly associated with maternal anemia and iron status. There was less supportive evidence of the involvement of nutritional factors such as consumption of iron-rich foods and HFI. Tobacco-using pregnant women were highly interested in receiving cessation services. There were missed opportunities for addressing tobacco use as part of antenatal care. Although antenatal clinics did not have a formal strategy to address tobacco use, there was interest among providers to add routine tobacco control services, if they were integrated with current practices such as services which address anemia and iron deficiency.

## References

[1] Mistry R, Dasika A (2018). Antenatal tobacco use and secondhand smoke exposure in the home in India. Nicotine Tob Res.

[2] Sinha DN, Suliankatchi RA, Amarchand R, Krishnan A (2016). Prevalence and sociodemographic determinants of any tobacco use and dual use in six countries of the WHO south-East Asia region: findings from the demographic and health surveys. Nicotine Tob Res.

[3] India Institute of Population Sciences and Macro International (2007). National Family Health Survey 3, 2005–06: India. Vol. 1.

[4] Maternal Health Division (2010). Guidelines for antenatal care and skilled attendance at birth.

[5] World Health Organization (2013). WHO recommendations for the prevention and management of tobacco use and second-hand smoke exposure in pregnancy. pp. 103.

[6] Subramoney S, Gupta PC (2008). Anemia in pregnant women who use smokeless tobacco. Nicotine Tob Res.

[7] Ganganahalli P, Pratinidhi A, Patil JA, Kakade SV (2015). Smokeless tobacco use & anaemia among pregnant women in Karad taluk western Maharashtra: A cross sectional study. Ntl J of Community Med.

[8] Ratsch A, Bogossian F (2014). Smokeless tobacco use in pregnancy: an integrative review of the literature. Int J Public Health.

[9] United States Department of Health and Human Services (2004). The health consequences of smoking: a report of the surgeon general.

[10] Klebanoff MA, Shiono PH, Selby JV, Trachtenberg AI, Graubard BI (1991). Anemia and spontaneous preterm birth. Am J Obstet Gynecol.

[11] Scholl TO, Hediger ML, Bendich A, Schall JI, Smith WK, Krueger PM (1997). Use of multivitamin/mineral prenatal supplements: influence on the outcome of pregnancy. Am J Epidemiol.

[12] Kocyigit A, Erel O, Gur S (2001). Effects of tobacco smoking on plasma selenium, zinc, copper and iron concentrations and related antioxidative enzyme activities. Clin Biochem.

[13] Northrop-Clewes CA, Thurnham DI (2007). Monitoring micronutrients in cigarette smokers. Clin Chim Acta.

[14] Chelchowska M, Ambroszkiewicz J, Gajewska J, Jablonska-Glab E, Maciejewski TM, Oltarzewski M (2016). Hepcidin and iron metabolism in pregnancy: correlation with smoking and birth weight and length. Biol Trace Elem Res.

[15] Blaha V, Yang ZJ, Meguid M, Chai JK, Zadak Z (1998). Systemic nicotine administration suppresses food intake via reduced meal sizes in both male and female rats. Acta Med (Hradec Kralove).

[16] Koopmann A, Bez J, Lemenager T, Hermann D, Dinter C, Reinhard I, Hoffmann H, Wiedemann K, Winterer G, Kiefer F (2015). Effects of cigarette smoking on plasma concentration of the appetite-regulating peptide ghrelin. Ann Nutr Metab.

[17] Miyata G, Meguid MM, Fetissov SO, Torelli GF, Kim HJ (1999). Nicotine's effect on hypothalamic neurotransmitters and appetite regulation. Surgery.

[18] Chaloupka FJ (2008). Smoking, food insecurity, and tobacco control. Arch Pediatr Adolesc Med..

[19] Cutler-Triggs C, Fryer GE, Miyoshi TJ, Weitzman M (2008). Increased rates and severity of child and adult food insecurity in households with adult smokers. Arch Pediatr Adolesc Med.

[20] Kim JE, Tsoh JY (2016). Cigarette smoking among socioeconomically disadvantaged young adults in association with food insecurity and other factors. Prev Chronic Dis.

[21] Shiffman S, West R, Gilbert D (2004). Recommendation for the assessment of tobacco craving and withdrawal in smoking cessation trials. Nicotine Tob Res.

[22] Anczak JD, Claire E (2003). Tobacco cessation in primary care: maximizing intervention strategies. Clin Med Res.

[23] Panda R, Persai D, Venkatesan S, Ahluwalia JS (2015). Physician and patient concordance of report of tobacco cessation intervention in primary care in India. BMC Public Health.

[24] Pati S, Patnaik S, Swain S (2014). 5A tobacco cessation strategy and physician's practice in Odisha, India: a cross-sectional study. Int J Prev Med.

[25] Pratinidhi A, Gandham S, Shrotri A, Patil A, Pardeshi S (2010). Use of ‘mishri’ a smokeless form of tobacco during pregnancy and its perinatal outcome. Indian J Community Med.

[26] Kaur J, Sachdeva KS, Modi B, Jain DC, Chauhan LS, Dave P, Singh RJ, Wilson N (2013). Promoting tobacco cessation by integrating'brief advice'in tuberculosis control programme. WHO South-East Asia J Public Health.

[27] Nair S, Schensul JJ, Begum S, Pednekar MS (2015). Use of smokeless tobacco by Indian women aged 18 – 40 years during pregnancy and reproductive years. PLoS One.

[28] Shelley D, Tseng T, Pham H, Nguyen L, Keithly S, Stillman F, Nguyen N (2014). Factors influencing tobacco use treatment patterns among vietnamese health care providers working in community health centers. BMC Public Health.

[29] Sarkar BK, West R, Arora M, Ahluwalia JS, Reddy KS, Shahab L (2017). Effectiveness of a brief community outreach tobacco cessation intervention in India: a cluster-randomised controlled trial (the BABEX trial). Thorax.

[30] Ram F, Lahir S, Parasuraman S, Singh LL, Paswan B, Singh SK, Das KC (2010). Global adult tobacco survey: India 2009-2010. Pp. 289.

[31] Bowen L, Bharathi AV, Kinra S, Destavola B, Ness A, Development ES (2012). Evaluation of a semi-quantitative food frequency questionnaire for use in urban and rural India. Asia Pac J Clin Nutr.

[32] Coates J, Swindale A, Bilinsky P. Household food insecurity access scale (HFIAS) for measurement of food access: Indicator guide (v3): Food and nutrition technical assistance project (FANTA). Washington DC.: Academy for Educational Development; 2007.

[33] Rutstein SO, Johnson K (2004). DHS comparitive reports 6: The DHS wealth index. Pp. 71.

[34] StataCorp. 12.1 edition (2012). College Station.

[35] Craney TA, Surles JG (2002). Model-dependent variance inflation factor cutoff values. Qual Eng.

[36] Miles MB, Huberman AM. Qualitative data analysis: an expanded sourcebook. Thousand Oaks: Sage; 1994.

[37] Strauss A, Corbin J (1990). Basics of qualitative research.

[38] Agaku IT, King BA (2014). Validation of self-reported smokeless tobacco use by measurement of serum cotinine concentration among US adults. Am J Epidemiol.

[39] Benowitz NL, Bernert JT, Caraballo RS, Holiday DB, Wang J (2009). Optimal serum cotinine levels for distinguishing cigarette smokers and nonsmokers within different racial/ethnic groups in the United States between 1999 and 2004. Am J Epidemiol.

